# Differential Regulation of the Phenazine Biosynthetic Operons by Quorum Sensing in *Pseudomonas aeruginosa* PAO1-N

**DOI:** 10.3389/fcimb.2018.00252

**Published:** 2018-07-23

**Authors:** Steven Higgins, Stephan Heeb, Giordano Rampioni, Mathew P. Fletcher, Paul Williams, Miguel Cámara

**Affiliations:** ^1^Centre for Biomolecular Science, School of Life Science, University of Nottingham, Nottingham, United Kingdom; ^2^Department of Plant and Microbial Biology, University of Zürich, Zurich, Switzerland; ^3^Department of Science, University Roma Tre, Rome, Italy

**Keywords:** *Pseudomonas aeruginosa*, phenazines, pyocyanin, quorum sensing, LasR, RhlR, RsaL, PqsE

## Abstract

The *Pseudomonas aeruginosa* quorum sensing (QS) network plays a key role in the adaptation to environmental changes and the control of virulence factor production in this opportunistic human pathogen. Three interlinked QS systems, namely *las, rhl*, and *pqs*, are central to the production of pyocyanin, a phenazine virulence factor which is typically used as phenotypic marker for analysing QS. Pyocyanin production in *P. aeruginosa* is a complex process involving two almost identical operons termed *phzA*_1_*B*_1_*C*_1_*D*_1_*E*_1_*F*_1_*G*_1_ (*phz1*) and *phzA*_2_*B*_2_*C*_2_*D*_2_*E*_2_*F*_2_*G*_2_ (*phz2*), which drive the production of phenazine-1-carboxylic acid (PCA) which is further converted to pyocyanin by two modifying enzymes PhzM and PhzS. Due to the high sequence conservation between the *phz1* and *phz2* operons (nucleotide identity > 98%), analysis of their individual expression by RNA hybridization, qRT-PCR or transcriptomics is challenging. To overcome this difficulty, we utilized luminescence based promoter fusions of each phenazine operon to measure in planktonic cultures their transcriptional activity in *P. aeruginosa* PAO1-N genetic backgrounds impaired in different components of the *las, rhl*, and *pqs* QS systems, in the presence or absence of different QS signal molecules. Using this approach, we found that all three QS systems play a role in differentially regulating the *phz1* and *phz2* phenazine operons, thus uncovering a higher level of complexity to the QS regulation of PCA biosynthesis in *P. aeruginosa* than previously appreciated.

**Importance:**

The way the *P. aeruginosa* QS regulatory networks are intertwined creates a challenge when analysing the mechanisms governing specific QS-regulated traits. Multiple QS regulators and signals have been associated with the production of phenazine virulence factors. In this work we designed experiments where we dissected the contribution of specific QS switches using individual mutations and complementation strategies to gain further understanding of the specific roles of these QS elements in controlling expression of the two *P. aeruginosa* phenazine operons. Using this approach we have teased out which QS regulators have either indirect or direct effects on the regulation of the two phenazine biosynthetic operons. The data obtained highlight the sophistication of the QS cascade in *P. aeruginosa* and the challenges in analysing the control of phenazine secondary metabolites.

## Introduction

*Pseudomonas aeruginosa* is a highly adaptable bacterium, which can be found in a range of challenging environments, including the human host. This is achieved in great part by the ability of this opportunistic pathogen to finely control the expression of a wide range of genes, including those involved in the production of virulence determinants, in response to environmental and metabolic stimuli (Lee et al., 2006; Balasubramanian et al., 2013; Sun et al., 2016). The expression of many virulence genes in *P. aeruginosa* is also controlled in a cell density dependent manner by quorum sensing (QS) (Smith and Iglewski, 2003; Bjarnsholt and Givskov, 2007).

*P. aeruginosa* has a sophisticated QS network consisting of three separate but interwoven systems, namely *las, rhl*, and *pqs* and their cognate QS signal molecules (QSMs). The QSMs *N*-3-oxo-dodecanoyl-homoserine lactone (3OC_12_-HSL) produced by LasI, and *N*-butanoyl-homoserine lactone (C_4_-HSL) produced by RhlI interact with their cognate transcriptional regulators LasR and RhlR respectively, leading to the activation or repression of multiple genes including the genes coding for their cognate signal synthases (Schuster et al., 2013). The LasR/3OC_12_-HSL complex also induces the transcription of *rsaL*, a gene integrated in the *las* QS system coding for the global transcriptional regulator RsaL (de Kievit et al., 1999). This protein directly represses the transcription of multiple genes, including *lasI*, hence exerting a homeostatic effect on 3OC_12_-HSL production, and conferring robustness to the expression of a sub-set of genes of the *las* regulon with respect to fluctuations in LasR levels (Rampioni et al., 2006, 2007; Bertani et al., 2007).

The *pqs* QS system is more complex than the *las* and *rhl* systems, since multiple enzymes encoded by the *pqsABCDE* operon are required for the synthesis of 2-alkyl-4(1*H*)-quinolones (AQs) including the QSMs 2-heptyl-4-hydroxyquinoline (HHQ), which in turn is converted to 2-heptyl-3-hydroxy-4-quinolone (PQS) by the monooxygenase PqsH. Both HHQ and PQS can bind to and activate the transcriptional regulator PqsR (also known as MvfR). The PqsR/HHQ and PqsR/PQS complexes bind the P*pqsA* promoter region and increase the transcription of the *pqsABCDE* operon, thus generating a feedback loop that accelerates AQ biosynthesis and increasing production of PqsE, coded by the last gene of the *pqsABCDE* operon (Heeb et al., 2011; Dulcey et al., 2013). PqsE is a thioesterase involved in AQ biosynthesis (Drees and Fetzner, 2015) but this protein also controls indirectly the expression of multiple virulence factors even in the absence of AQs. The molecular mechanism by which PqsE impacts on QS target gene expression remains unknown (Hazan et al., 2010; Rampioni et al., 2010, 2016).

The QS circuit of *P. aeruginosa* has been widely reported to have a hierarchal structure. Under growth conditions using rich media, it is generally accepted that the *las* QS system is the first to become active leading to the activation of the *rhl* and *pqs* systems (Pesci et al., 1997; de Kievit et al., 2002; Gallagher et al., 2002; Xiao et al., 2006). However it has been reported that RhlR can in part overcome the absence of the *las* system in late stationary phase (Dekimpe and Deziel, 2009). RhlR is required for production of certain virulence factors but has a negative impact on the *pqs* system by repressing PQS signal production through interference with the expression of *pqsR* and *pqsABCDE* (McKnight et al., 2000; Wade et al., 2005; Xiao et al., 2006; Brouwer et al., 2014). In turn the *pqs* system has a positive effect upon the *rhl* system, as addition of PQS to a *P. aeruginosa* culture has been shown to increase the levels of RhlR and the *rhl* QS signal C_4_-HSL (McKnight et al., 2000; Diggle et al., 2003). The interactions of the QS systems are detailed in Figure S1.

QS has been shown to affect the transcription of hundreds of downstream genes (Schuster et al., 2003; Wagner et al., 2003; Rampioni et al., 2007, 2010) with some of these specifically controlled by distinct QS systems, while others are induced or repressed by multiple QS regulators (Schuster and Greenberg, 2007; Farrow et al., 2008; Cornforth et al., 2014; Rampioni et al., 2016).

The production of pyocyanin (PYO), a key virulence factor produced by *P. aeruginosa*, has been linked to multiple QS systems. This particular phenazine is often used as a marker to assess QS behavior as it is easily measurable and contributes significantly toward the green color of *P. aeruginosa* cultures (Frank and Demoss, 1959). Although PYO is the most studied phenazine in *P. aeruginosa*, this organism is capable of producing up to 5 different phenazine derivatives (Mavrodi et al., 2001, 2010). Phenazine biosynthesis begins with the conversion of chorismic acid to phenazine-1-carboxylic acid (PCA) by the action of the enzymes encoded by the biosynthetic operon *phzABCDEFG*, which is conserved across the fluorescent Pseudomonad species (Mavrodi et al., 2006, 2010). Interestingly *P. aeruginosa* has 2 functional copies of this operon designated *phz1* and *phz2*. Both operons produce PCA, which can be further converted to phenazine-1-carboxamide by the action of PhzH and to 1-hydroxyphenazine by PhzS. The action of PhzM is to convert PCA to 5-methylphenazine-1-carboxylic acid betaine, which can be further converted to PYO by PhzS (Mavrodi et al., 2001, 2006, 2010).

PYO production has been linked to QS in many reported studies and to date LasR, RhlR, RsaL, PqsE, PqsR and both AQ signal molecules HHQ and PQS have been found to play a role in the control of its production (Whiteley and Greenberg, 2001; Gallagher et al., 2002; Diggle et al., 2003; Schuster et al., 2003; Wagner et al., 2003; Rampioni et al., 2007, 2010; Farrow et al., 2008; Lu et al., 2009; Liang et al., 2011; Recinos et al., 2012; Cabeen, 2014; Sun et al., 2017). Although QS controls PYO production, the high sequence conservation between the two phenazine producing operons *phz1* and *phz2* have made analysing their individual expression by DNA hybridization techniques challenging (Schuster et al., 2003; Wagner et al., 2003; Rampioni et al., 2007, 2010).

It is unlikely that both phenazine biosynthesis operons are controlled in the same manner as they are located some distance apart on the PAO1 chromosome and have very different promoter regions (Mavrodi et al., 2001; Whiteley and Greenberg, 2001; Rampioni et al., 2007; Winsor et al., 2011). The *phz1* operon (from PA4210 to PA4216) is flanked by *phzM* upstream (PA4209) and *phzS* downstream (PA4217), both of which are required to produce PYO. The *phz2* operon (from PA1899 to PA1905) is flanked upstream by the *qscR* gene (PA1898), coding for the orphan QS receptor QscR, and downstream by the PA1906 gene, coding for a hypothetical protein of unknown function. The *phzH* gene (PA0051) is unlinked to the other phenazine biosynthetic genes (Figure S2). It would appear by looking at the positions of the operons on the chromosome of *P. aeruginosa* PAO1 that the *phz1* operon is clustered with the genes required to produce PYO, and hence could be more closely associated with PYO production than *phz2*. That said, the *phz2* operon has been shown to contribute significantly to the production of PYO, especially under non-planktonic growth conditions (Recinos et al., 2012; Dietrich et al., 2013).

There is a greater quantity of available information about the control of *phz1* than *phz2*, and a *lux* box, for LasR or RhlR binding, has been predicted upstream of the −10 region of the *phz1* promoter (P*phzA1*) (Whiteley and Greenberg, 2001). The QS repressor RsaL has also been shown to bind to this promoter in an electrophoretic mobility shift assay (EMSA) at the downstream end of the −10 promoter region, thus acting as a repressor of *phz1* transcription (Rampioni et al., 2007). Moreover, RsaL exerts an indirect negative effect on *phz1* transcription by increasing the production of the *phz1* repressor protein CdpR (Sun et al., 2017).

Less is known about the regulation of the *phz2* promoter (P*phzA2*). The intergenic region between *qscR* and *phz2* was probed for RsaL binding by different groups with negative results (Rampioni et al., 2007; Sun et al., 2017), hence RsaL appears to control P*phzA1* but not P*phzA2*. Recinos and colleagues found that *phz2* transcription is induced by HHQ under anaerobic conditions (Recinos et al., 2012). Identification of a predicted ANR/DNR binding site within the P*phzA2* promoter supports the notion that *phz2* transcription is increased in anaerobic environments (Trunk et al., 2010). The orphan *luxR* QS regulator, QscR, which is encoded directly upstream of *phzA2* has been reported to be a repressor of *phzA2* (Ledgham et al., 2003; Lequette et al., 2006). The *qscR* and *phz2* intergenic region was probed for QscR binding with a negative result (Lee et al., 2006) suggesting the effect of QscR on *phzA2* is indirect due to the ability of QscR to form inactive heterodimers with LasR and RhlR (Chugani et al., 2001).

To gain a further understanding of the control of each phenazine biosynthesis operon by QS, *lux-*based promoter fusions for each operon were created and tested in a range of QS mutants. We identified an RsaL dependent switch, which can move PCA production from one operon to the other, and *vice versa*. This switching mechanism was confirmed by modification of the QS network activity in selected mutants with the addition of QS signal molecules and specific QS regulator genes expressed from plasmids. This allowed us to confirm the hierarchal structure of QS in rich media under planktonic conditions and to develop a more in depth model of how QS controls *phz1* and *phz2* transcription in *P. aeruginosa*.

## Materials and methods

### Bacterial strains and growth conditions

The bacterial strains and plasmids used in this study are detailed in Table S1. They were routinely grown in Lysogeny Broth (LB) at 37°C with shaking at 200 rpm, with the exception of *P. aeruginosa* conjugation recipient strains, which were incubated at 42°C. When required, LB was supplemented with the following antibiotics: for *E. coli*, 10 μg ml^−1^ tetracycline (Tc), 30 μg ml^−1^ chloramphenicol (Cm), or 100 μg ml^−1^ ampicillin (Ap); for *P. aeruginosa*, 150 μg ml^−1^ Tc, 375 μg ml^−1^ Cm or 800 μg ml^−1^ streptomycin (Sm). Media were supplemented with 1 mM (final concentration) isopropyl β-D-1-thiogalactopyranoside (IPTG) for inducible strains where required, unless otherwise stated. Synthetic signal molecules PQS and 2-methyl-3-hydroxy-4-quinolone (mPQS) were added to cultures at a final concentration of 100 μM where required. To select for *P. aeruginosa* after mating experiments LB agar plates were supplemented with 15 μg ml^−1^ nalidixic acid (Nal).

### DNA manipulations

All plasmids generated and/or used in this study are listed in Table S1. Routine DNA manipulations including extraction, restriction, ligation, electroporation, conjugation and agarose gel electrophoresis were performed using standard molecular methods (Sambrook and Russell, 2001). Plasmid extraction was completed using a Qiagen™ QiaQuick miniprep kit following the manufacturer's instructions. The Tc^R^ marker of pMINI-CTX1 derived constructs integrated into the chromosome of *P. aeruginosa* was removed using the Flp recombinase system as previously described (Hoang et al., 1998, 2000). All primers used for DNA amplification by PCR are detailed in Table S2. DNA sequencing was conducted at the University of Nottingham's DNA sequencing facility.

### Generation of pP*phzA1*-lux, pP*phzA2*-lux, and pRsal plasmids

pMINI-lux was generated by cloning the *Bam*HI-*Eco*RI fragment of pBluelux (Atkinson et al., 2008), containing the *luxCDABE* operon, into similarly digested mini-CTX1, using standard molecular methods (Sambrook and Russell, 2001). The P*phzA1* and P*phzA2* promoter regions were PCR amplified from *P. aeruginosa* PAO1 chromosomal DNA using primer pairs FWP*phzA1*-RVP*phzA1*, and FWP*phzA2*-RVP*phzA2*, respectively (Table S2). The PCR products were independently cloned into the pMINI-lux construct between the *Eco*RI and *Xho*I restriction sites, resulting in the plasmids pP*phzA1*-lux and pP*phzA1*-lux, respectively.

The *rsaL* coding region was amplified by PCR from *P. aeruginosa* PAO1 chromosomal DNA using primers FW*rsaL* and RV*rsaL* (Table S2). The resulting PCR product was cloned into pME6032 between the *Eco*RI and *Xho*I restriction sites using standard molecular techniques. This plasmid was introduced to *P. aeruginosa* strains by electroporation (Choi et al., 2006).

All cloned fragments obtained by PCR were verified by DNA sequencing to match the reference sequences (Winsor et al., 2011).

### Generation of *P. aeruginosa* mutant strains

To generate the double mutant strain *P. aeruginosa pqsE*ind Δ*lasR*, the *lasR* gene was deleted from the chromosome of the PAO1 *pqsE*ind strain (Rampioni et al., 2010) by using the pME3087-Δ*lasR* plasmid (Harrison et al., 2014).

Briefly, the pME3087-Δ*lasR* plasmid was mobilized by conjugation into the *P. aeruginosa pqsE*ind recipient strain using *E. coli* S17.1 λ*pir* as a donor. Exconjugants were selected on LB plates supplemented with 150 μg ml^−1^ Tc and 15 μg ml^−1^ Nal. Strains were re-streaked twice on LB lacking antibiotic and then subjected to 1 round of Tc sensitivity enrichment to select for double cross-over events (Voisard et al., 1994). Five colonies which were Tc^S^ were then tested by PCR for loss of the *lasR* coding region.

To generate a *P. aeruginosa* PAO1 mutant strain with an *rsaL* deletion (Δ*rsaL*), allelic exchange was obtained by using the pDM4-Δ*rsaL* plasmid, derived from the suicide vector pDM4 (Milton et al., 1996). The upstream and the downstream DNA regions of *rsaL* were PCR amplified from *P. aeruginosa* PAO1 chromosomal DNA using primer pairs FW*rsaL*UP + RV*rsaL*UP and FW*rsaL*DOWN + RV*rsaL*DOWN, respectively (Table S2). The upstream and downstream PCR fragments were subsequently cloned in pDM4 by XhoI-BamHI and BamHI-XbaI restriction, respectively. The resulting pDM4-Δ*rsaL* plasmid was verified by restriction analysis and sequencing. Allelic exchange in *P. aeruginosa* PAO1 following conjugal mating with the *E. coli* S17.1 λ*pir* (pDM4-Δ*rsaL*) donor strain and sucrose counter selection was performed as previously described (Westfall et al., 2004). The resulting PAO1 Δ*rsaL* mutant strain was confirmed by PCR.

### Gene expression assays

Three independent single colonies of *P. aeruginosa* strains carrying reporter constructs were grown overnight in LB (separate tubes) at 37°C with shaking at 200 rpm. One-milliliter of overnight culture was washed in 1 ml of fresh LB to remove secreted bacterial products and QS signal molecules. Twenty-microliters aliquots were inoculated into 1 ml of fresh LB, and 300 μl of the resulting cultures were dispensed into wells of a 96-well black flat-transparent-bottom microtiter plate. When needed, strains with inducible genes were grown with or without 1 mM IPTG unless otherwise stated. Microtiter plates were incubated at 37°C in a TECAN GENios automated luminometer-spectrophotometer with which luminescence and turbidity were recorded every 30 min. Promoter activity per cell is given as relative light units divided by absorbance at 600 nm wavelength (A_600_).

### Statistical tests

Standard deviation of the mean of the three biological replicates is reported. A paired *t*-test was used to compare each mutant with the relevant control for reach experiment. A *P*-value of ≤0.05 was considered significant.

## Results

### QS control of *phz1* and *phz2* expression

To ascertain how the phenazine operons *phz1* and *phz2* are regulated by the different elements of the QS circuit in *P. aeruginosa* PAO1-N, the pP*phzA1*-lux and pP*phzA2*-lux reporter plasmids, containing transcriptional fusions between the P*phzA1* and P*phzA2* promoter regions and the *luxCDABE* operon for bioluminescence, respectively, were generated and inserted in the chromosome of strain PAO1-N and different mutants derived from it. In detail, in the pP*phzA1*-lux plasmid a 727-bp DNA fragment comprising the entire intergenic region between *phzM* and *phzA1* was cloned upstream of the *luxCDABE* operon, while in pP*phzA2*-lux a 497-bp DNA fragment including the entire intergenic region between *qscR* and *phzA2* was cloned instead. By including these relatively large promoter regions the likelihood of missing any key regulatory element upstream of the known unique transcription start sites of P*phzA1* and P*phzA2* (Dötsch et al., 2012) was minimized. Cloned promoter regions include the first two codons of *phzA1* and *phzA2*, respectively. Since the vast majority of studies on the QS circuit of *P. aeruginosa* have been undertaken in rich media, LB was used in this work so that predictions about the behavior of the QS network in specific QS mutants could be made and the results obtained compared with previous studies.

**Figure 1:**
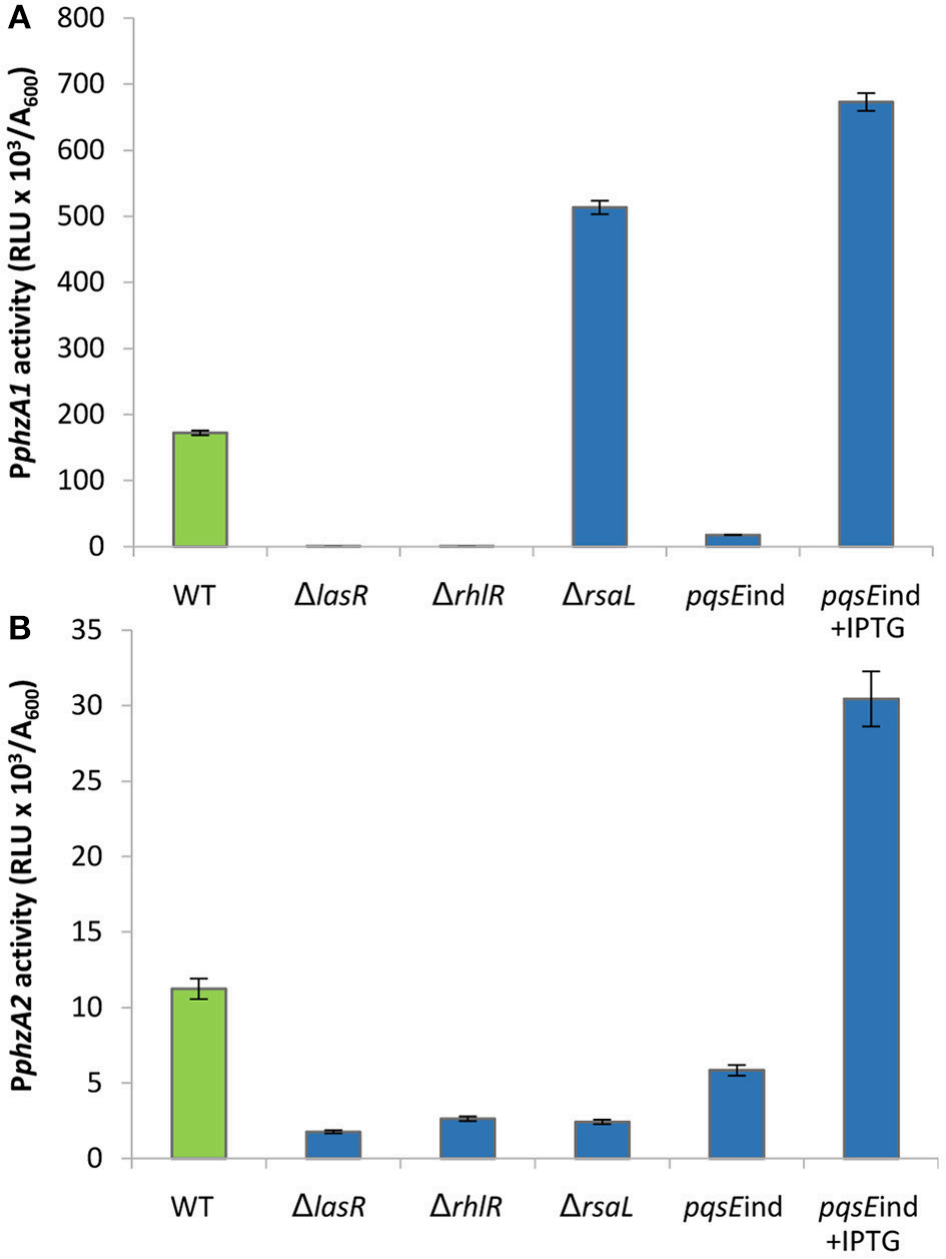
Effect of QS elements on P*phzA1* and P*phzA2* activity. Maximal promoter activity per cell measured in the indicated strains derived from *P. aeruginosa* PAO1-N carrying the transcriptional fusions P*phzA1*-*lux* **(A)** or P*phzA2*-*lux* **(B)**. Bioluminescence (relative light units, RLU) and cell density (A_600_) were recorded after 6 h incubation at 37°C. Mean of three independent experiments is shown with standard deviation.

Firstly, the impact of QS elements, previously identified as key players in the regulation of PYO production, on P*phzA1* and P*phzA2* activity was investigated in PAO1-N, since there have been some strain-specific differences shown in the regulation of these operons by QS (Sun et al., 2016). Figure 1, shows that under the planktonic conditions studied the activity of P*phzA1* is several fold higher than that of P*phzA2* which is in line with what has been detected in *P. aeruginosa* PA14 (Recinos et al., 2012). LasR, RhlR, and PqsE showed a positive effect on the activation of the P*phzA1* and P*phzA2* promoters. P*phzA1* activity was completely abrogated in the Δ*lasR* and Δ*rhlR* mutants, and strongly decreased (90% reduction) in the non-induced *pqsE* conditional mutant strain, *pqsE*ind (*P* < 0.01). The effect of LasR, RhlR, and PqsE on P*phzA2* appear to be milder although still significant (*P* < 0.05), with reporter activities reduced by 78, 71, and 52% in the Δ*lasR*, Δ*rhlR* and non-induced *pqsE*ind strains, respectively. When PqsE was fully induced with 1 mM IPTG in the *pqsE*ind strain, a 3.5-fold increase in promoter activity of both P*phzA1* and P*phzA2* was observed relative to the PAO1-N wild type (*P* < 0.01). Conversely, RsaL had an opposite effect on the two promoters, since P*phzA1* activity is significantly increased (298% increase) and P*phzA2* activity decreased (80% reduction) (*P* < 0.01), in the Δ*rsaL* mutant compared to PAO1-N wild type. These results are in accordance with published data for other *P. aeruginosa* strains, showing a positive effect of LasR, RhlR and PqsE on PCA biosynthesis in the human pathogen *P. aeruginosa* PA14 and in the rhizosphere bacterium *P. aeruginosa* PA1201 (Recinos et al., 2012; Sun et al., 2016, 2017). Also the dual effect of RsaL on P*phzA1* and P*phzA2* is in line with what was previously observed in *P. aeruginosa* PA1201 (Sun et al., 2017). The growth data for this experiment is shown in Figure S3.

**Figure 2:**
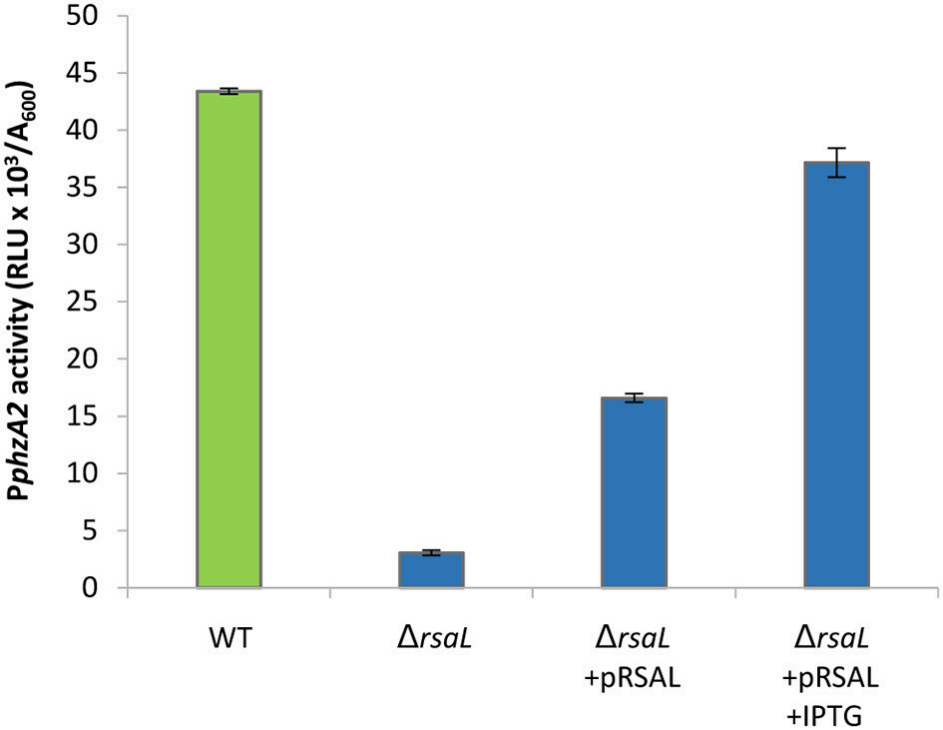
Effect of pRsaL on P*phzA2* activity. Maximal promoter activity of P*phzA2*-*lux* in the wild type PAO1-N and the *rsaL* mutant background, with and without complementation. Promoter activities are normalized by cell density (A_600_).

To further validate the regulation of P*phzA2* by RsaL, the *rsaL* deletion was complemented *via* the IPTG inducible pRsaL plasmid. Some partial restoration of P*phzA2* activity was observed in the Δ*rsaL* strain in the presence of pRsaL, likely as a consequence of basal *rsaL* transcription from the *tac* promoter (Guzman et al., 1995), while in the presence of 0.1 mM IPTG P*phzA2* activity was restored to wild type levels (*P* < 0.05) (Figure 2). Overall, these data confirm that in PAO1-N RsaL is a repressor of *phz1* transcription and has a positive effect upon P*phzA2*, the latter likely mediated by an ancillary P*phzA2*-regulator under the control of RsaL, since purified RsaL has not been shown to directly bind to P*phzA2* in EMSA studies (Rampioni et al., 2007; Sun et al., 2017). The growth data for this experiment is shown in Figure S4.

### Detailed analysis of the impact of the QS cascade on P*phzA1* activity

**Figure 3:**
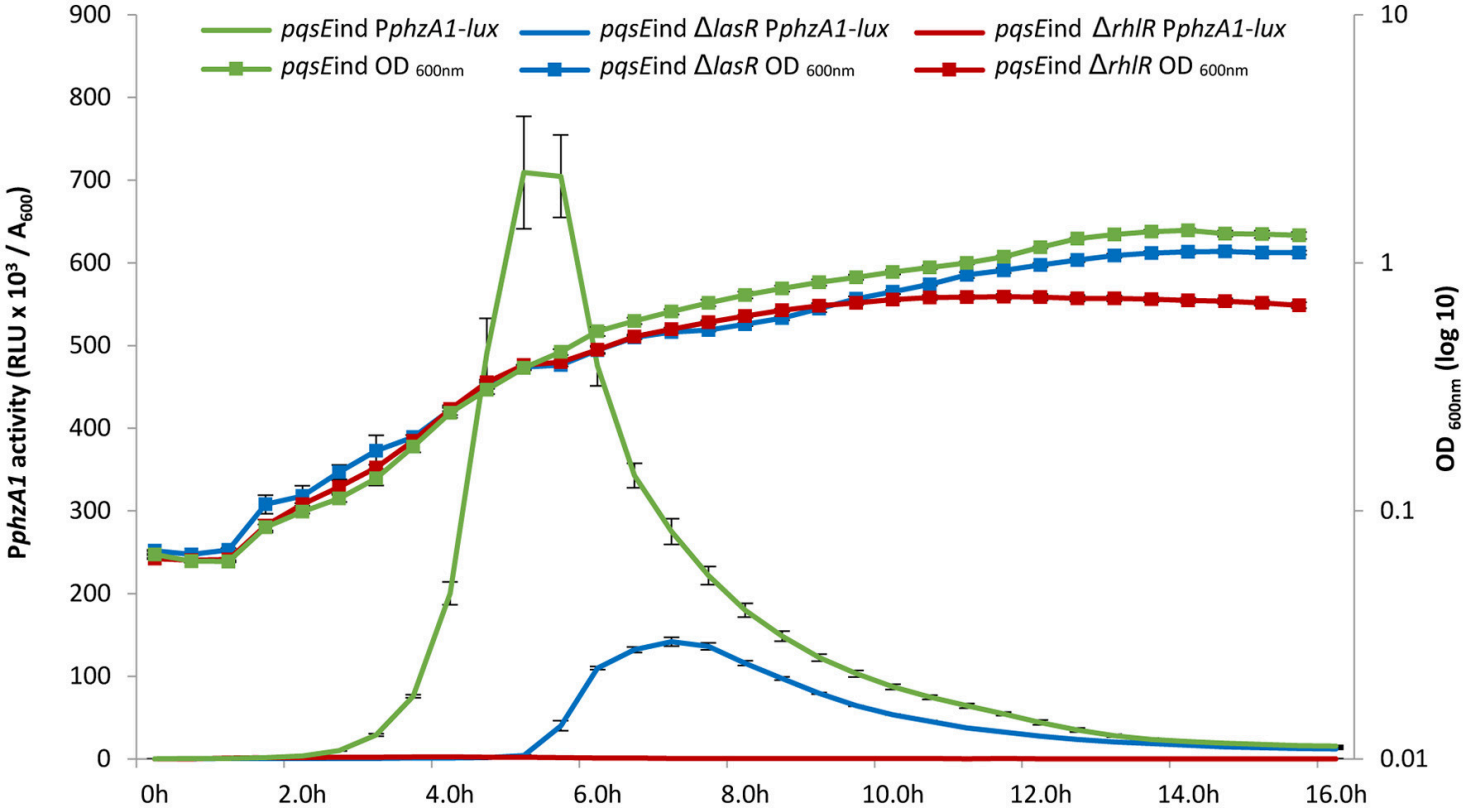
A delay in the timing of P*phzA1* activation was observed when *lasR* was mutated in the *pqsE*ind strain. The *pqsE*ind strain increased the activity of P*phzA1*-*lux* (green line) but when *lasR* is mutated a 2 h delay in promoter activation is observed (blue line) compared with the *pqsE*ind. When *rhlR* is deleted from the *pqsE*ind strain P*phzA1*-*lux* activity is abolished (red line). The growth curves of the 3 mutants are also shown and this data is plotted on the Z axis. All strains were induced with 1 mM IPTG and promoter activities are normalized by cell density (A_600_).

High levels of PqsE resulted in an increase in promoter activity for both phenazine biosynthesis operons (Figure 1). Since a *lux-*box is present in the P*phzA1* promoter region and PqsE does not act as a transcriptional regulator, it can be hypothesized that PqsE exerts a positive effect on P*phzA1* activity *via* the LasR and/or RhlR transcriptional regulators. This hypothesis was tested by analysing P*phzA1* activity in the double mutants *pqsE*ind Δ*lasR* and *pqsE*ind Δ*rhlR* respectively in which *pqsE* expression can be restored in the presence of IPTG. Figure 3 reveals that while PqsE induction with IPTG resulted in high P*phzA1* activity in the *pqsE*ind strain, the activity of this promoter under induced conditions was reduced by 80% in the *pqsE*ind Δ*lasR* mutant and a 2 h delay in activation of P*phzA1* relative to the *pqsE*ind strain induced with IPTG was observed. P*phzA1* activity was completely abrogated in the *pqsE*ind Δ*rhlR* background. The growth data for this experiment is shown in Figure S5.

**Figure 4:**
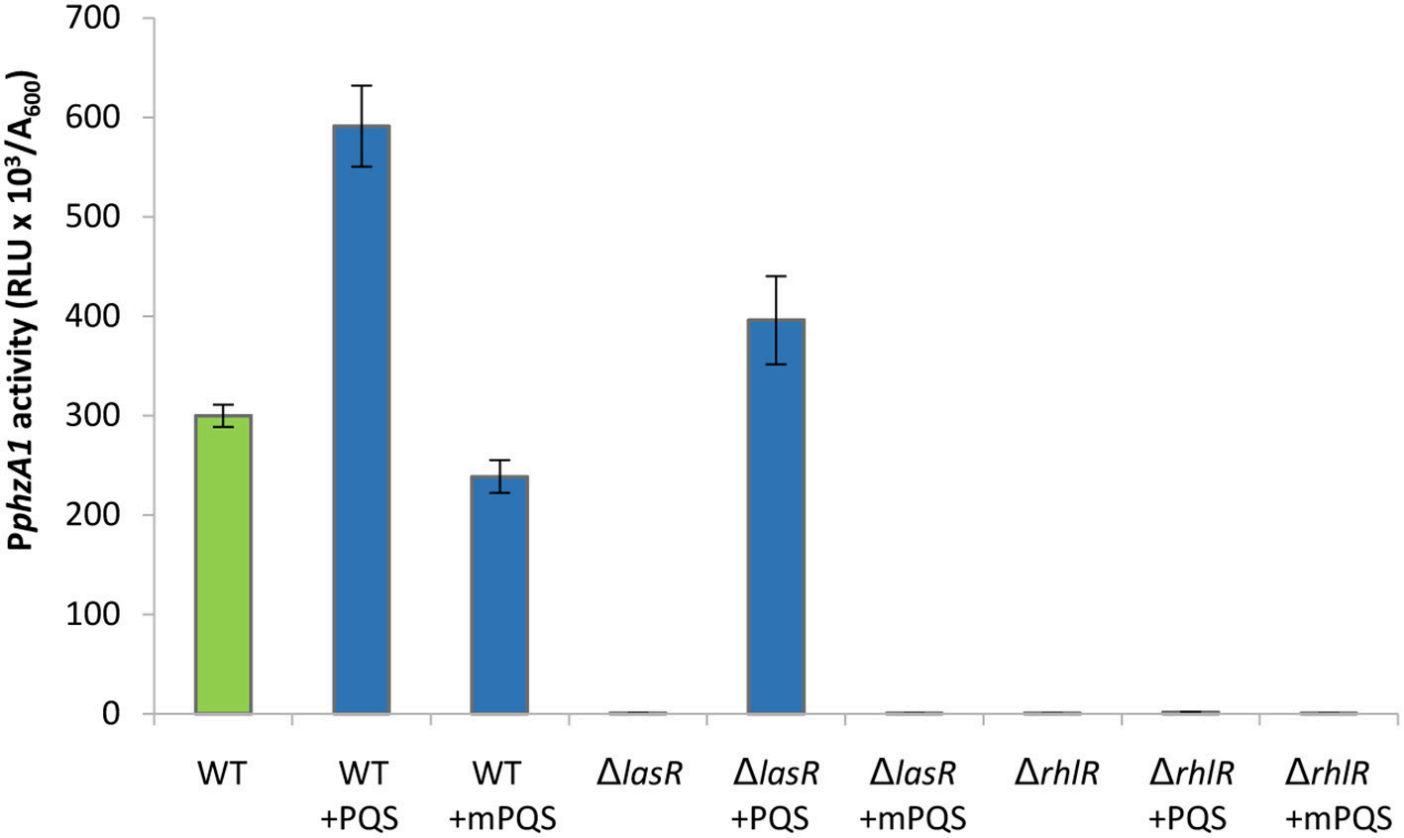
The addition of exogenous PQS compensates for a mutation in *lasR* by increasing activity of P*phzA1*. Maximal promoter activity of P*phzA1*-*lux* in the wild type PAO1-N, *lasR* and *rhlR* deletion backgrounds in the presence of PQS or methyl PQS (mPQS). Promoter activities are normalized by cell density (A_600_).

The *las* QS system has a positive effect on the activity of both the *rhl* and *pqs* QS systems (Pesci et al., 1997; Medina et al., 2003; Xiao et al., 2006). Moreover, a study by McKnight et al. (2000) showed that addition of exogenous PQS positively regulates the *rhl* system, and Diggle et al. (2003) showed that addition of exogenous PQS advances and enhances pyocyanin production and increases RhlR levels. We therefore hypothesized that the reduction of transcriptional activity of P*phzA1* in the *pqsE*ind Δ*lasR* mutant could be caused by reduced activity of the *rhl* and *pqs* systems in this mutant background, and hence exogenous provision of PQS should compensate for a *las* mutation. To test this, P*phzA1* activity was analyzed in the Δ*lasR* and Δ*rhlR* strains in the presence of 100 μM exogenous PQS. To discard any effects related to the iron chelating properties of PQS, the non-signaling quinolone molecule methyl PQS (mPQS) was used as a control, since this molecule is capable of binding iron like PQS, but is unable to trigger gene expression *via* PqsR (Diggle et al., 2007). Addition of PQS was found to compensate for a *lasR* deletion (*P* < 0.05), while an *rhlR* deletion resulted in no activation of the P*phzA1* promoter, irrespective of the absence or presence of PQS (Figure 4). The addition of PQS also impacted on the timing of P*phzA1* gene expression in both wild type and *lasR* deletion strains with the activation of this promoter triggered 1 h earlier than in the absence of this signal molecule (data not shown). The growth data for this experiment is shown in Figure S6.

These data suggest that RhlR is a direct positive regulator of the *phz1* operon, as loss of this element abolishes P*phzA1* activity, while LasR acts as an indirect P*phzA1* regulator.

Addition of PQS in the previous experiment would be expected to increase the expression of the *pqsABCDE* operon and hence PqsE production through the activation of PqsR. The data presented here suggests that PqsE is required to activate P*phzA1* and PqsE would be present in high levels after the addition of exogenous PQS to the culture. To further investigate the importance of RhlR and LasR in the PqsE-mediated activation of P*phzA1* the above experiments were repeated using the *pqsE*ind conditional mutant with additional *lasR* and *rhlR* mutations.

**Figure 5:**
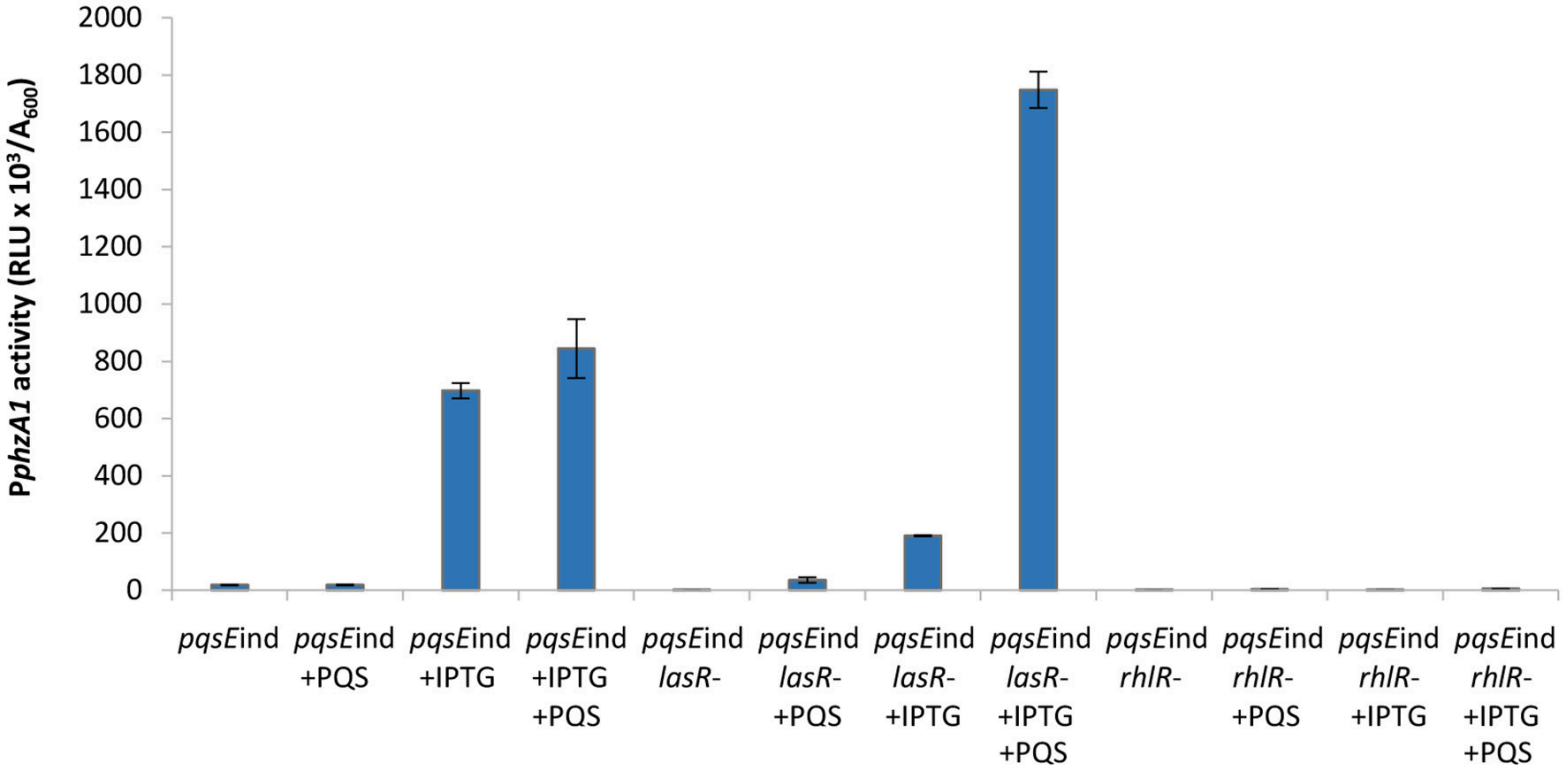
PQS is unable to induce high levels of P*phzA1* transcription in the absence of PqsE or RhlR. Maximal promoter activity of P*phzA1*-*lux* in the *pqsE*ind strain and *pqsE*ind strain carrying additional *lasR* or *rhlR* mutations in the presence or absence of IPTG and PQS. Promoter activities are normalized by cell density (A_600_).

Addition of PQS to the un-induced *pqsE*ind strain resulted in no significant increase in P*phzA1* activity, compared to the un-induced *pqsE*ind strain (*P* < 0.05). This result confirms that PqsE rather than PQS on its own or through PqsR activation is required to reach high levels of P*phzA1* transcription (Figure 5). Since PqsR has been reported to directly bind to *rhlI/R* resulting in some increased expression of these genes (Maura et al., 2016) this result suggests that activated RhlR in combination with PqsR are unable to activate P*phzA1* transcription. In the *pqsE*ind strain the expression of *pqsE* is decoupled from that of *pqsABCD*, due to a transcriptional terminator introduced downstream of *pqsD*, hence PqsE production is not under the control of PqsR (Rampioni et al., 2010). Addition of PQS when *pqsE*ind was induced by IPTG slightly increased P*phzA1* activity compared with the non-induced *pqsE*ind without PQS, this may be due to some PqsR direct activation of *rhlI/R*. Again PQS was unable to trigger reporter gene expression in the *pqsE*ind Δ*lasR* strain when *pqsE* was not induced. Interestingly, when *pqsE* was fully induced and PQS was added to the *pqsE*ind Δ*lasR* mutant, a significant increase in P*phzA1* activity (*P* < 0.05) of approximately 50% was observed compared with the fully induced *pqsE*ind mutant. No P*phzA1* expression was detected in the *pqsE*ind Δ*rhlR* strain under any of the conditions tested confirming the importance of RhlR in activating *phz1* transcription (Figure 5). The growth data for this experiment is shown in Figure S6.

The high levels of P*phzA1* activity observed when both, PQS is added and PqsE expression is induced with IPTG in the *pqsE*ind Δ*lasR* strain, could be due to low levels of RsaL, which is a repressor of P*phzA1* (Figure 1). Since LasR activates the *rsaL* promoter (P*rsaL*), the Δ*lasR* mutant is expected to express low levels of RsaL. Overall, these data are consistent with PqsE and RhlR as the key activators of the *phz1* operon with RsaL acting as a repressor.

### Detailed analysis of the impact of the QS cascade on P*phzA2* activity

**Figure 6:**
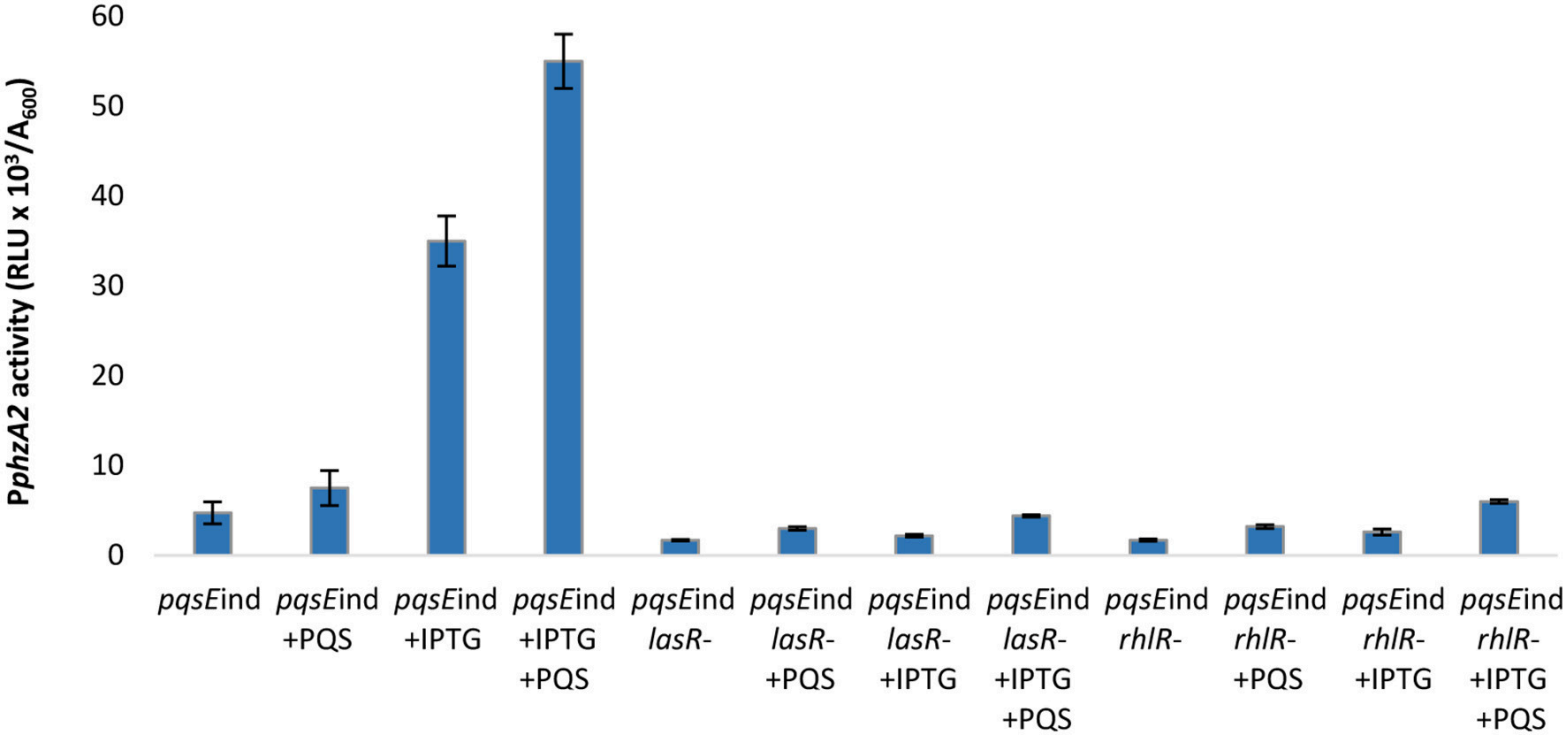
PQS is unable to induce P*phzA2* transcription in the absence of PqsE, LasR, and RhlR. Maximal promoter activity of P*phzA1*-*lux* in the *pqsE*ind strain and *pqsE*ind strain carrying additional *lasR* or *rhlR* mutations in the presence or absence of IPTG and PQS. Promoter activities are normalized by cell density (A_600_).

**Figure 7:**
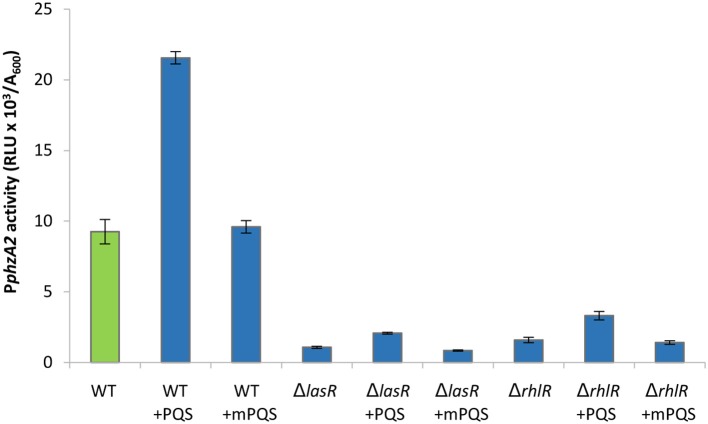
P*phzA2* activity cannot be restored by exogenous PQS in *lasR* or *rhlR* mutants. Maximal promoter activity of P*phzA2*-*lux* in the wild type PAO1-N, *lasR* and *rhlR* deletion backgrounds in the presence of PQS or methyl PQS (mPQS). Promoter activities are normalized by cell density (A_600_).

In Figure 1 we show that both PqsE and RsaL exert positive control over the expression of P*phzA2* but the influence the *las* and *rhl* systems may have on this regulation is not clear. To investigate this further, the expression of P*phzA2* was studied in *pqsE*ind and *pqsE*ind with a *lasR* or a *rhlR* deletion. When *pqsE* was induced in either strain, P*phzA2* activity could only achieve 5% and 10% of the *pqsE*ind strain (Figure 6), growth data Figure S7. This differed from the result obtained for P*phzA1*, as a *lasR* deletion in the *pqsE*ind induced strain decreased but did not abrogate P*phzA1* activity (Figure 5). To ascertain whether addition of exogenous PQS could compensate for *lasR* and *rhlR* deletions, P*phzA2* activity was evaluated in the Δ*lasR* and Δ*rhlR* mutants supplemented with exogenous PQS or mPQS, the latter molecule used as an iron-binding negative control as before. Unlike P*phzA1*, where PQS restored promoter activity in the Δ*lasR* mutant, no significant increase (*P* < 0.05) in P*phzA2* activity was observed when PQS was added to the Δ*lasR* and Δ*rhlR* strains (Figure 7), growth data Figure S8.

These results suggests that both LasR and RhlR are required to activate P*phzA2*. Since addition of exogenous PQS increases the levels of PqsE (Heeb et al., 2011) we next investigated whether PqsE or PqsR were responsible for the increase in P*phzA2* activity. The same experimental strategy as for P*phzA1* analysis was used and activity of P*phzA2* was assayed in the *pqsE*ind mutant strain and the *pqsE*ind strains with additional *lasR* and *rhlR* deletions, in the presence of exogenously added PQS and IPTG.

The result of this experiment showed that addition of PQS to the un-induced *pqsE*ind strain resulted in no significant increase in P*phzA2* activity, compared to the un-induced *pqsE*ind strain (*P* < 0.05). When *pqsE* was induced with IPTG, P*phzA2* activity was triggered and further increased by addition of exogenous PQS (*P* < 0.05) (Figure 6). These data suggest that PqsE rather than PqsR is required to positively regulate P*phzA2*, as it was for P*phzA1*. Hardly any increase in the expression of the P*phzA2* promoter was observed in the *pqsE*ind strains carrying additional *lasR* and *rhlR* deletions, either in the absence or presence of IPTG and/or PQS, suggesting that LasR, RhlR and PqsE are all key for P*phzA2* transcription.

We have shown that RsaL has a positive effect on P*phzA2* (Figures 1, 2). The *rsaL* promoter is positively regulated by LasR, hence in the *lasR* deletion mutant low levels of RsaL would be expected, which in turn should have a negative impact on P*phzA2* activity. Therefore, we hypothesized that LasR is an indirect activator of P*phzA2* acting *via* RsaL, and to test this we transformed the *lasR* mutant strain with the inducible pRsaL plasmid. As LasR affects the activity of the *rhl* and *pqs* QS systems, exogenous PQS was also added to increase the activity of the *rhl* and *pqs* QS systems.

**Figure 8:**
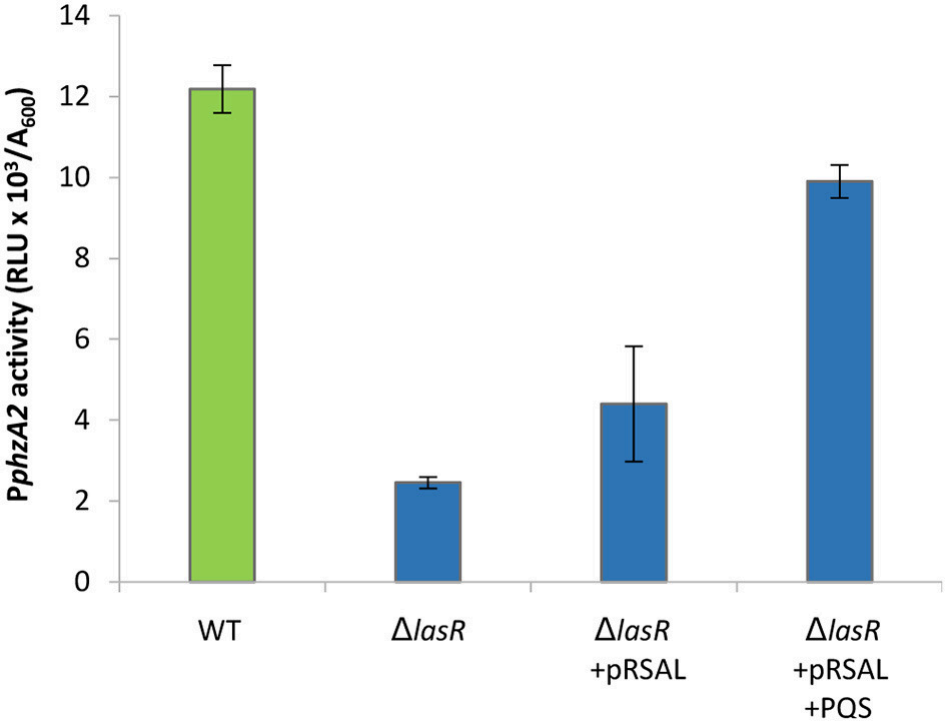
P*phzA2* activity in a *lasR* mutant is restored by overexpressing RsaL and supplementing with exogenous PQS. Maximal promoter activity of P*phzA2*-*lux* in the wild type PAO1-N, *lasR* and *lasR* deletion carrying pRsaL in the presence of PQS. Promoter activities are normalized by cell density (A_600_).

The result of these experiments suggest that LasR is an indirect activator of P*phzA2*, since an increase in P*phzA2* activity in the *lasR* mutant carrying pRsaL was observed, compared with the *lasR* mutant. Addition of exogenous PQS further increased P*phzA2* activity to the level of the wild type PAO1-N level when pRsaL was induced with IPTG (*P* < 0.05) (Figure 8), growth data Figure S9.

**Figure 9:**
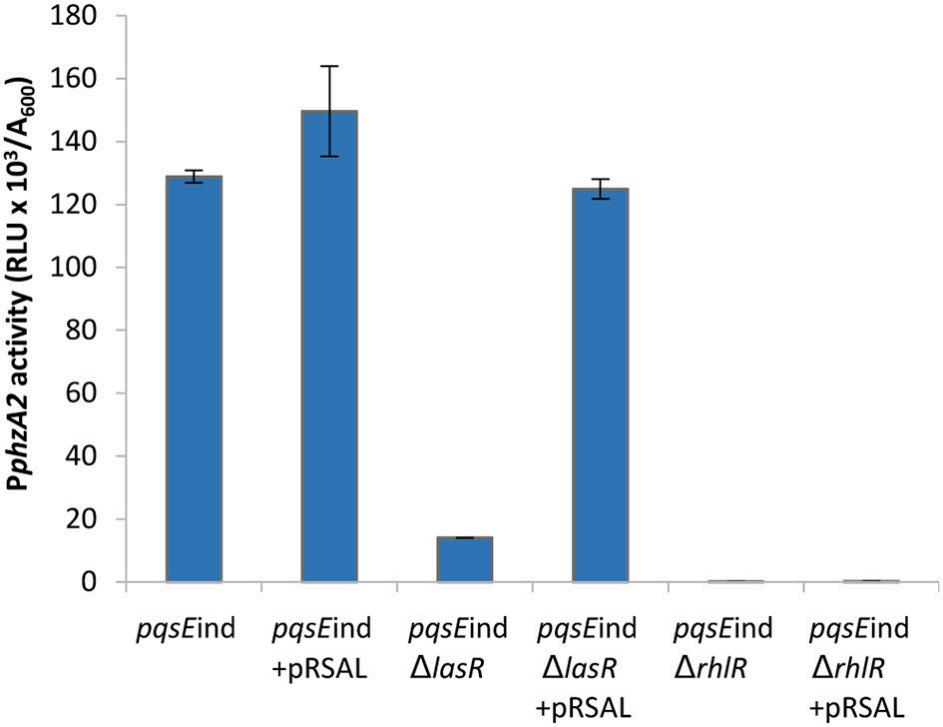
RsaL can activate P*phzA2* in the absence of LasR when *pqsE* is induced and RhlR is present. Maximal promoter activity of P*phzA2*-*lux* in the *pqsE*ind strain and *pqsE*ind strain with additional *lasR* or *rhlR* mutations and in the presence or absence of pRsaL. All strains were induced with 0.1 mM IPTG. Promoter activities are normalized by cell density (A_600_).

These data suggest that RsaL alone is unable to induce the P*phzA2* promoter to wild type levels and must be working in conjunction with other regulatory elements. To gain further evidence that LasR is an indirect activator of P*phzA2* and investigate the requirement of PqsE and RhlR to activate P*phzA2*, we introduced pRsaL in the *pqsE*ind and *pqsE*ind strains with additional *lasR* and *rhlR* deletions. When RsaL and PqsE expression was induced in these strains with IPTG, P*phzA2* activity of the *pqsE*ind Δ*lasR* strain carrying pRsaL was significantly increased (*P* < 0.05) compared to the *pqsE*ind Δ*lasR* strain and P*phzA2* activity was comparable to the induced *pqsE*ind strain. No P*phzA2* activity was observed in the *pqsE*ind strain carrying an additional *rhlR* deletion, confirming that RsaL, RhlR, and PqsE are all required to trigger transcription of the *phz2* operon (Figure 9). The growth data for this experiment is shown in Figure S10.

## Discussion

### New model of phenazine production control by QS

Here it has been demonstrated that the QS regulators LasR, RhlR, RsaL and the enzyme PqsE are all involved in controlling the expression of both phenazine operons *phz1* and *phz2* in *P. aeruginosa* with some differences. Initially it was unclear which regulators played a direct role in activating each operon and which were indirect because of the hierarchal structure of the QS network in rich media (Figure 1). A combination of the deletion of specific genes, the inducible expression of specific QS regulators and/or exogenous provision of QS signal molecules has allowed us to tease out which regulators are direct activators and which can be considered indirect because of their effect upon the QS network (Figures 3–9). Although rich media is not representative of the natural environment in which *P. aeruginosa* is found our experiments have closed an unanswered question of which QS regulators directly control each *phz* operon.

**Figure 10:**
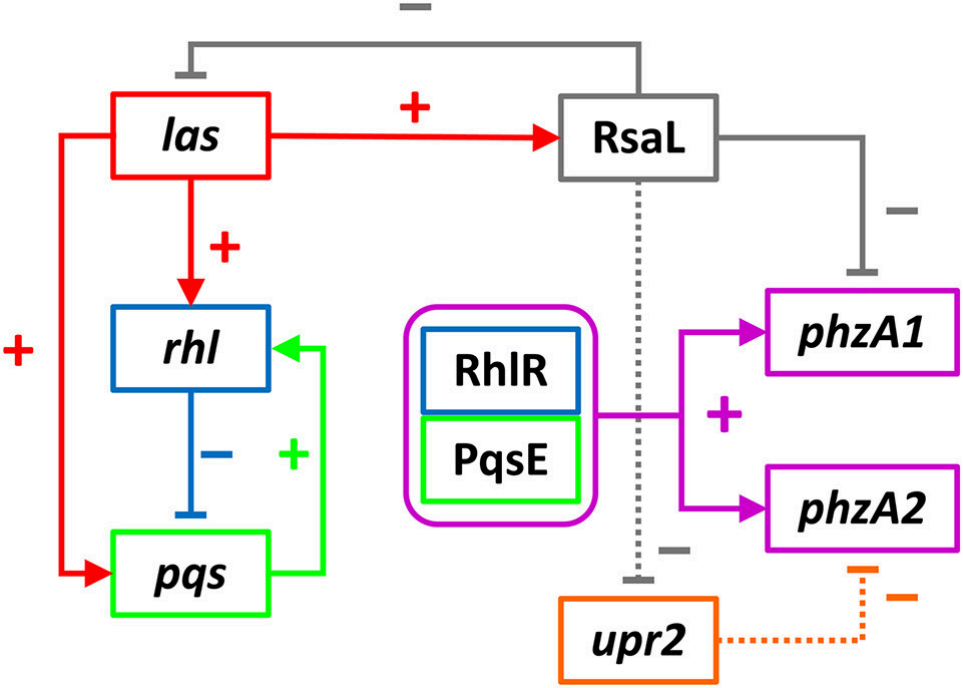
Interactions of the QS cascade to control P*phzA1* and P*phzA2* activity. The auto-inducing *las* system positively regulates the *rhl* and *pqs* system via its regulator LasR, as well as the QS repressor RsaL. The *rhl* system represses the *pqs* system, which in turn has a positive effect on the *rhl* system. These two systems interact via RhlR and PqsE (purple box) which are both required to induce P*phzA1* and P*phzA2*. The QS repressor RsaL is a repressor of P*phzA1* but an indirect activator of P*phzA2*. We hypothesize that this is achieved by its ability to repress the promoter of an Unknown Phenazine Regulator of *phzA2* (orange box), that remains to be identified. Through this mechanism production of PCA from *phz1* can be switched to *phz2* and vice versa. Positive interactions are indicated by arrows whereas negative interactions are indicated by T-bars.

The results obtained allow us to postulate a model by which the QS cascade interacts and controls phenazine production in planktonic cultures (Figure 10).

Evidence has been presented showing that RhlR is a positive regulator for both operons and also that PqsE must be present to induce each operon. This is not surprising as it has been previously reported that PqsE and RhlR are both required for pyocyanin production (Farrow et al., 2008). It could be the case that all genes in the *rhl* regulon may be co-dependent upon PqsE as the production of the RhlR controlled genes *lasB* and *rhlA* are enhanced in the presence of PqsE (Farrow et al., 2008; Rampioni et al., 2010). The data presented show that PqsE rather than PqsR is required to activate the transcription of both phenazine biosynthesis operons. It was demonstrated by Recinos et al. (2012) that HHQ plays a role in activation of P*phzA2* under anaerobic conditions. HHQ would inevitably increase the levels of PqsE as *pqsABCDE* is a direct target for PqsR when bound to either HHQ or PQS (Fletcher et al., 2007; Rampioni et al., 2016). As molecular oxygen is required to convert HHQ to PQS (Schertzer et al., 2010) it would appear that under anaerobic conditions PqsE can still be produced when the signal HHQ binds PqsR, emphasizing that PqsE is important for activating the phenazine operons under both aerobic and anaerobic conditions.

In rich media, LasR drives expressions of the *rhl* and *pqs* systems, which then interact through RhlR and PqsE to activate the phenazine operon promoters. A deletion of *lasR* caused a reduction in P*phzA1* activity, which further demonstrates that in rich media the QS cascade has a hierarchal structure. This is in accordance with the work of others who have demonstrated that the loss of LasR results in a delay in production of pyocyanin (Dekimpe and Deziel, 2009; Cabeen, 2014). LasR also drives expression from P*rsaL* and in turn RsaL represses P*phzA1*, P*lasI* and its own production. *In vitro* protein-DNA interaction experiments revealed that RsaL binds to the P*phzA1* promoter at a region encompassing the −10 sequence (Rampioni et al., 2007; Sun et al., 2017), hence RsaL directly exerts a repressive effect on *phz1* expression. Moreover, RsaL was shown to exert an indirect repressive effect on P*phzA1* by increasing the expression of the P*phzA1*-repressor CdpR (Sun et al., 2017). It has been hypothesized by Rampioni et al. (2007) that RsaL maintains signal homeostasis by repressing P*lasI* and in the context of phenazine production could provide a similar feature (Bondi et al., 2017). RsaL could act to keep P*phzA1* inactive until the *rhl* and *pqs* systems are interacting before commencing transcription, thereby creating a checkpoint in the system. The QS signal molecule PQS has multiple functions as it can bind iron and also act as an anti-oxidant (Diggle et al., 2007; Häussler and Becker, 2008). In the presence of oxygen, pyocyanin generates reactive oxygen species (ROS) (Rada and Leto, 2013). Hence this checkpoint could function to allow adequate PQS to be produced and reduce deleterious effects of ROS produced by pyocyanin before triggering transcription of *phz1*.

It is thought that RsaL has a secondary function and strong evidence that RsaL can repress P*phzA1* but indirectly induce P*phzA2* has been presented. Previous studies have failed to demonstrate an interaction between RsaL and a DNA probe encompassing the P*phzA2* promoter region (Rampioni et al., 2007; Sun et al., 2017), suggesting that the positive effect exerted by RsaL on *phz2* expression is not direct. RsaL increases the expression of the P*phzA1*-repressor CdpR, but a ChIP-seq assay did not show any interaction between CdpR and the P*phzA2* promoter region in strain PA1201 (Zhao et al., 2016), suggesting that CdpR is not involved in the RsaL-mediated activation of P*phzA2*. The positive effect of RsaL on P*phzA2* is probably achieved *via* an unidentified phenazine biosynthesis gene regulator, which we termed Unidentified Phenazine Regulator of *phzA2* (Upr2). Although this regulator has not been identified, data presented thus far strongly imply the existence of this additional regulator, which in turn is controlled by the QS repressor RsaL.

We hypothesize that Upr2 is a P*phzA2* repressor and its expression could be repressed by RsaL. If Upr2 had a positive effect upon P*phzA2* we would expect that in an *rhlR* mutant some P*phzA2* activity would be observed. We show that when RsaL and PqsE are present but *rhlR* is mutated, no P*phzA2* activity was observed (Figure 9) making it unlikely that Upr2 is a positive regulator. It is likely that when the QS network is activated by LasR, that RsaL represses the promoter of *upr2* and as Upr2 is turned over and diluted through cell division that P*phzA2* can be triggered by RhlR in conjunction with PqsE, since both the *rhl* and *pqs* systems are positively regulated by LasR. In these experiments we observed significantly less activity from P*phzA2* compared with P*phzA1*, further suggesting that the P*phzA2* promoter is tightly controlled by a repressor. Upr2 could have its own regulon which may have significant overlap with that of the *las* regulon. We found that *phz2* transcription can be triggered in a *lasR* mutant when the *rsaL* deletion is complemented and PQS added exogenously to stimulate RhlR and PqsE production (Figure 8). Hence it is likely that some of the genes identified as *lasR-* or *rsaL-* specific in comparative transcriptome studies could belong to the *upr2* regulon.

We hypothesize that *P. aeruginosa* can switch PCA production from *phz1* to *phz2* when RsaL levels are elevated and from *phz2* to *phz1* when RsaL is absent. This switch could be related to a reduction in oxygen availability and an increase in oxidative stress as the population size increases, however, this remains to be investigated.

PCA is converted to PYO *via* the action of PhzM and PhzS. The *phzM* gene is located directly upstream of *phzA1* and the intergenic region between these has two predicted *lux* boxes (Whiteley and Greenberg, 2001). The *lux* boxes are flanked by two *rsaL* binding sites (Rampioni et al., 2007) which suggests that *phz1* and *phzM* are controlled in a similar manner. In the study by Rampioni et al. (2007) a microarray assay was used to identify the *rsaL* regulon. In that work it was discovered that both *phzM* and *phzS* were up regulated in the *rsaL* mutant compared with the wild type, suggesting that RsaL represses both genes. Here we present evidence that RsaL also represses P*phzA1*. As *phzM, phzS*, and *phz1* are all required to produce PYO, which in turn contributes toward oxidative stress. It is conceivable that when oxidative stress is high, RsaL can repress *phz1, phzM* and *phzS* but maintain PCA production by indirectly activating *phz2*. Evidence to support this hypothesis was provided when the oxidative stress response regulator OxyR was shown to bind the promoter of *rsaL* (Wei et al., 2012). A previous study of the OxyR regulon showed that when this regulator is mutated, pyocyanin levels increase, suggesting that OxyR can repress pyocyanin production and this could be achieved through RsaL (Vinckx et al., 2010).

One of the proposed main functions of phenazines is to cycle electrons which allows *P. aeruginosa* to continue respiration in microaerophilic environments by controlling the intracellular redox state (Dietrich et al., 2013). Switching off PYO production would therefore cause a problem under these conditions. Unlike PYO, PCA can donate electrons to iron (III) rather than oxygen, hence maintaining redox homeostasis without producing ROS (Wang and Newman, 2008; Wang et al., 2011). PYO may be used in addition to PCA to cycle electrons as O_2_ is a better electron accepter than iron. Through this switch, phenazine production may continue while lowering oxidative stress on the bacterium and maintaining redox balance.

## Author contributions

SHi, SHe, GR, MF, PW, and MC designed the study and analyzed the data. SHi, GR, and MF conducted the experiments. SHi, SHe, GR, and MC wrote the manuscript. All authors reviewed the manuscript.

### Conflict of interest statement

The authors declare that the research was conducted in the absence of any commercial or financial relationships that could be construed as a potential conflict of interest.
