# Supplementary material for: Differential Regulation of the Phenazine Biosynthetic Operons by Quorum Sensing in Pseudomonas aeruginosa PAO1-N
Source: Front Cell Infect Microbiol. 2018 Jul 23;8:252. doi: 10.3389/fcimb.2018.00252 (PMC6064868; doi:10.3389/fcimb.2018.00252)
Supplement: Supplementary file 1 [file Data_Sheet_1.PDF]

Supplementary material

Differential regulation of the phenazine biosynthetic operons by quorum sensing in *Pseudomonas aeruginosa* PAO1-N

Steven Higgins[1,2], Stephan Heeb[1], Giordano Rampioni[1,3], Mathew P. Fletcher[1], Paul Williams[1], Miguel Cámara [1*]

[*] **Corresponding author** Miguel Cámara: miguel.camara@nottingham.ac.uk

Table S1. Bacterial strains and plasmids generated and/or used in study

Strain / Plasmid	Relevant Characteristics	Reference / Source
E. coli		
S17.l λ*pir*	*thi pro hsdR hsdM_ recA* RP4-2-Tc::Mu-Km::Tn*7* λ*pir*	(Grant et al., 1990)
DH5α	F⁻ *ϕ*80d*lacZ* ΔM15 Δ(*lacZYA-argF*)*U169 recA1 hsdRI7* (r^-_k m^+_k) *supE44 λ⁻ thi-1 relA1 gyrA96*	(Hanahan, 1983)
P. aeruginosa		
PAO1-N	Nottingham collection wild type *P. aeruginosa* PAO1 strain.	
*pqsE*ind	PAO1-N conditional mutant in which the expression of *pqsE* is inducible with IPTG.	(Rampioni et al., 2010)
Δ*lasR*	PAO1-N mutant with *lasR* in frame deletion.	(Harrison et al., 2014)
Δ*rhlR*	PAO1-N mutant with *rhlR* in frame deletion.	(Rampioni et al., 2010)
Δ*rsaL*	PAO1-N mutant with *rsaL* in frame deletion.	This study
*pqsE*ind Δ*lasR*	PAO1-N mutant with *lasR* in frame deletion in which the expression of *pqsE* is inducible with IPTG.	This study
*pqsE*ind Δ*rhlR*	PAO1-N mutant with *rhlR* in frame deletion in which the expression of *pqsE* is inducible with IPTG.	(Rampioni et al., 2010)
**Plasmid**		
mini-CTX1	Site-specific integration vector for *P. aeruginosa*; Tc^R.	(Hoang et al., 2000)
pBluelux	pBluescript II SK+ derived cloning vector containing the *luxCDABE* operon; Ap^R.	(Atkinson et al., 2008)
pDM4	Suicide vector; *sacBR*; oriR6K; Cm^R.	(Milton et al., 1996)
pFLP2	Source of Flp recombinase: Ap^R.	(Hoang et al., 1998)
pME6032	P*tac* pVS1/p15A shuttle expression vector; Tc^R.	(Heeb et al., 2002)
pME3087-Δ*lasR*	pME3087 derived mutagenesis plasmid containing *lasR* upstream and downstream regions to generate a *lasR* in frame deletion; Tc^R.	(Harrison et al., 2014)
pDM4-Δ*rsaL*	pDM4 derived mutagenesis plasmid containing *rsaL* upstream and downstream regions to generate a *rsaL* in frame deletion: Cm^R.	This study
pRsaL	pME6032 derived plasmid for IPTG-inducible expression of *rsaL*; Tc^R.	This study
pMINI-lux	mini-CTX1 derivative containing the promoterless *luxCDABE* operon: Tc^R.	This study
pP*phzA1*-lux	pMINI-lux derived plasmid carrying the P*phzA1*::*luxCDABE* transcriptional fusion to monitor P*phzA1* activity; Tc^R.	This study
pP*phzA2*-lux	pMINI-lux derived plasmid carrying the P*phzA2*::*luxCDABE* transcriptional fusion to monitor P*phzA2* activity; Tc^R.	This study

Table S2

Oligonucleotides (5'-3') used during this study, restriction sites are underlined and start codon highlighted yellow.

Oligonucleotide	Sequence
FW P*phzA1* (*Xho*I)	TATCTCGAGCAGCAGCAAGATTCGAATTATT
RV P*phzA1* (*Eco*RI)	TATGAATTCGTT**CAT**GCGCCGCCTCCG
FW P*phzA2* (*Xho*I)	TATCTCGAGGCTCAACTGAATCGACGCCT
RV P*phzA2* (*Eco*RI)	TATGAATTCTCG**CAT**GGTGCGAATCTCC
FW *rsaL* (*Eco*RI)	TATGAATTC**ATG**GCTTCACACGAGAGAAC
RV *rsaL* (*Xho*I)	TATCTCGAGTTACTCTCTGATCTTGCCTCT
FW*rsaL*UP (*Xho*I)	CCGCTCGAGCGCATCGCCTCCAGCGT
RV*rsaL*UP (*Bam*HI)	TATGGATCCGTGTGAAGC**CAT**TGCTCTG
FW*rsaL*DOWN (*Bam*HI)	TATGGATCCTTGCATTTCTATATAGAAGG
RV*rsaL*DOWN (*Xba*I)	TGCTCTAGACTGGGAACCGTCCATCTAC

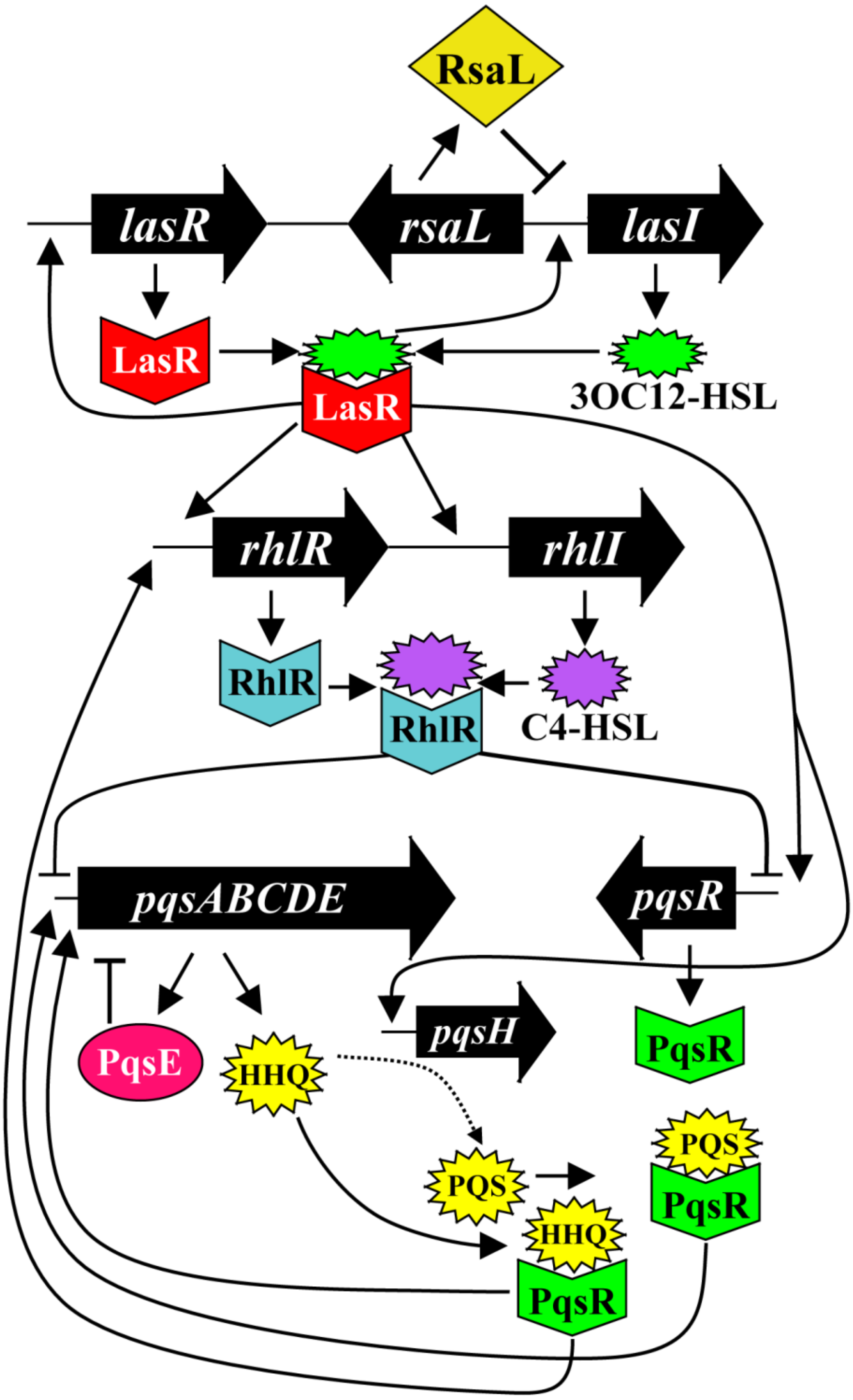

Supplementary Figure S1. Interaction between the *las*, *rhl* and *pqs* QS systems of *P. aeruginosa*.

It is generally accepted that in rich growth medium the *las* system is the first QS system to become active. LasR is able to positively influence *rhlI*, *rhlR*, *pqsR* and *pqsH* promoter activity and as a result the *rhl* and *pqs* systems become active. The *rhl* system represses the *pqs* system as RhlR is able to repress *pqsR* and *pqsABCDE* promoter activity. The *pqs* system has a positive effect upon the *rhl* system, as addition of PQS to a *P. aeruginosa* culture has been shown to increase the levels of RhlR and the *rhl* QS signal C_4-HSL. Arrows represent positive interactions whereas T-bars represent repression.

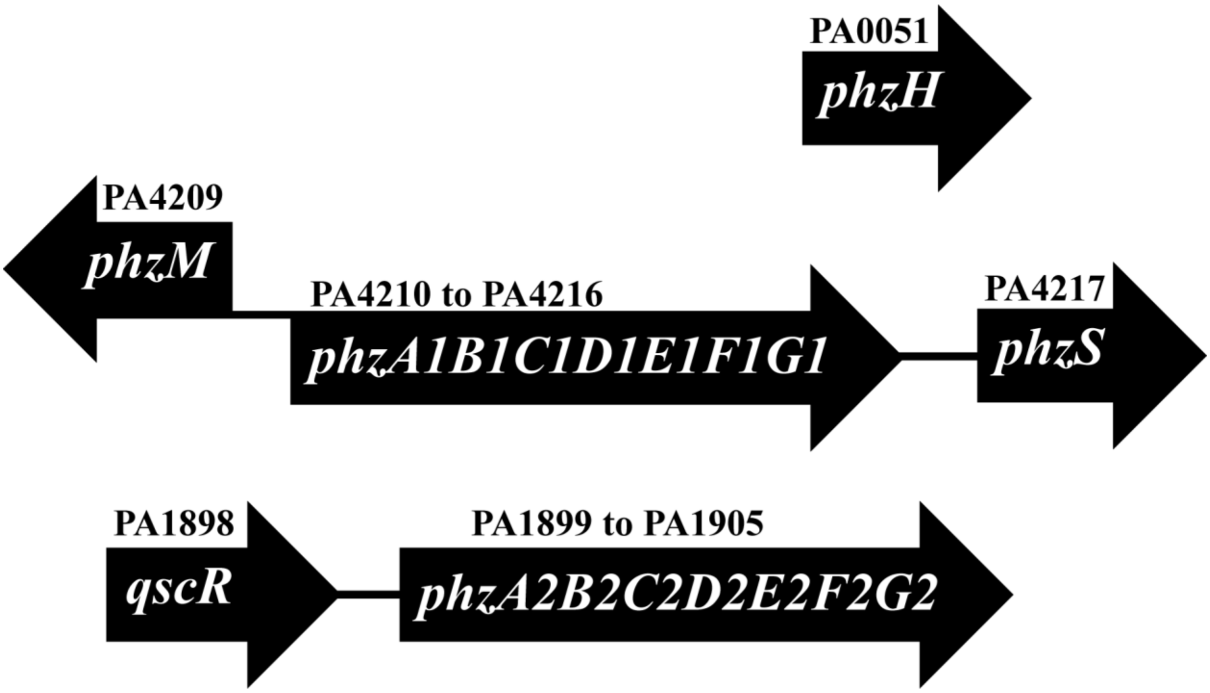

Supplementary Figure S2. The layout of the phenazine genes in the PAO1 chromosome
The phenazine biosynthetic operon *phz1* is flanked upstream by *phzM* and downstream by *phzS*. Approximately 2,000 open reading frames (ORF's) separate the *phz1* operon from the *phz2* operon, which is flanked upstream by the orphan QS homologue *qscR*. Another 2,000 ORF's separate *phz2* from the final phenazine gene *phzH*, which is located close to the origin of replication.

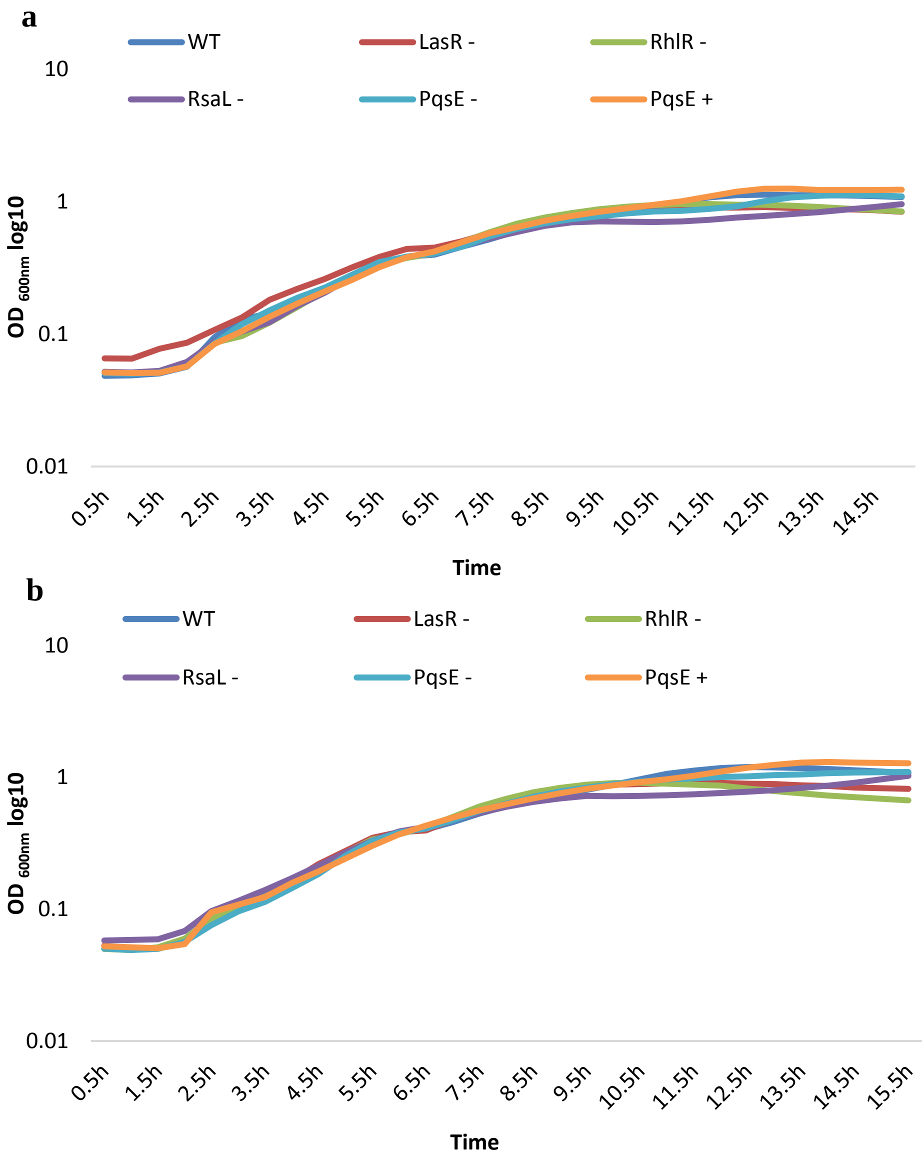

Supplementary Figure S3 – Growth dynamics of the strains from Figure 1a (top) and 1b (bottom). Mean of 3 biological replicates is shown.

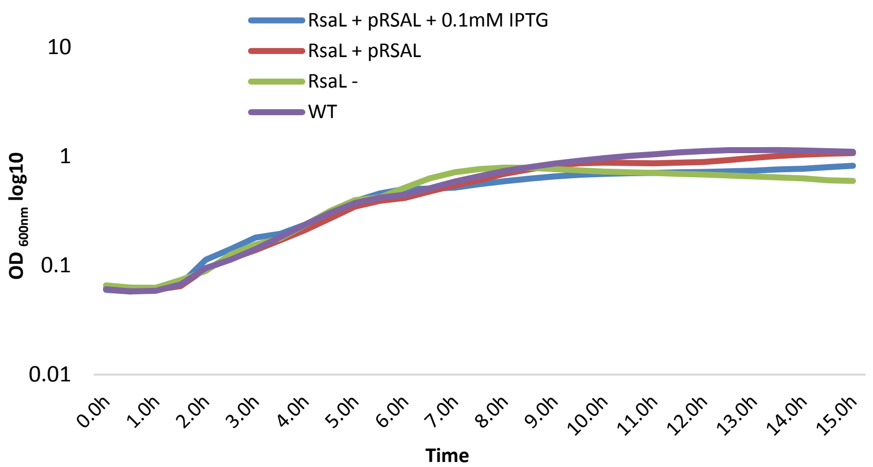

Supplementary Figure S4 – Growth dynamics of the strains from Figure 2 Mean of 3 biological replicates is shown.

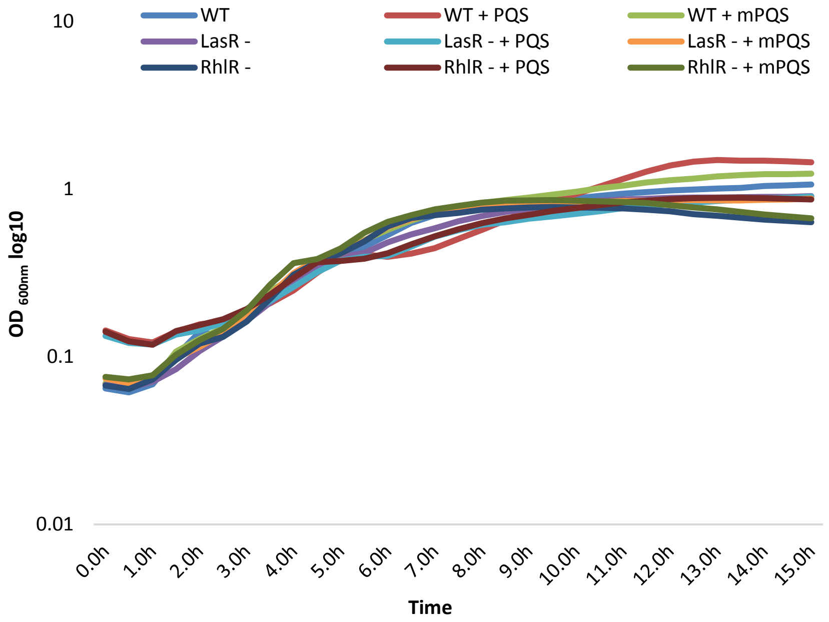

Supplementary Figure S5 – Growth dynamics of the strains from Figure 4 Mean of 3 biological replicates is shown.

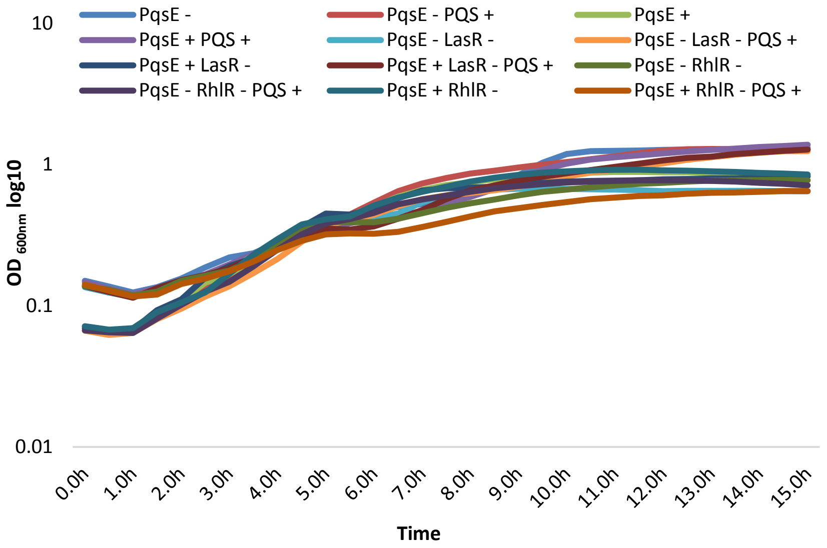

Supplementary Figure S6 – Growth dynamics of the strains from Figure 5. Mean of 3 biological replicates is shown.

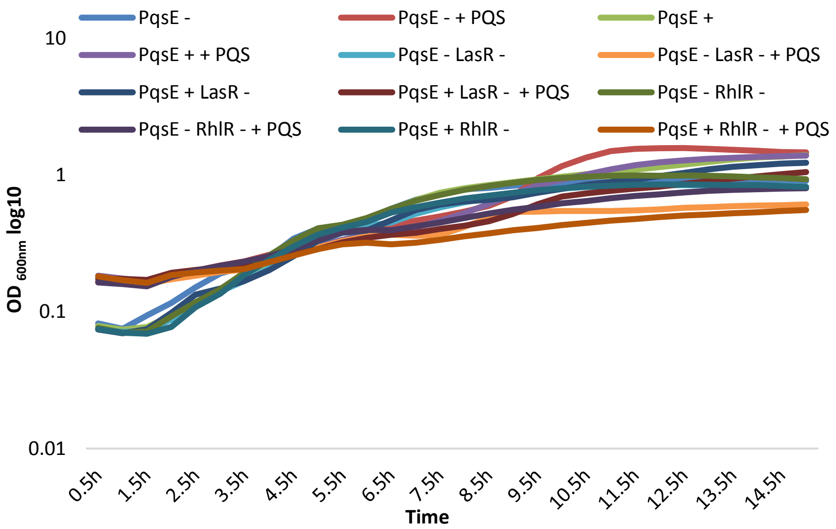

Supplementary Figure S7 – Growth dynamics of the strains from Figure 6. Mean of 3 biological replicates is shown.

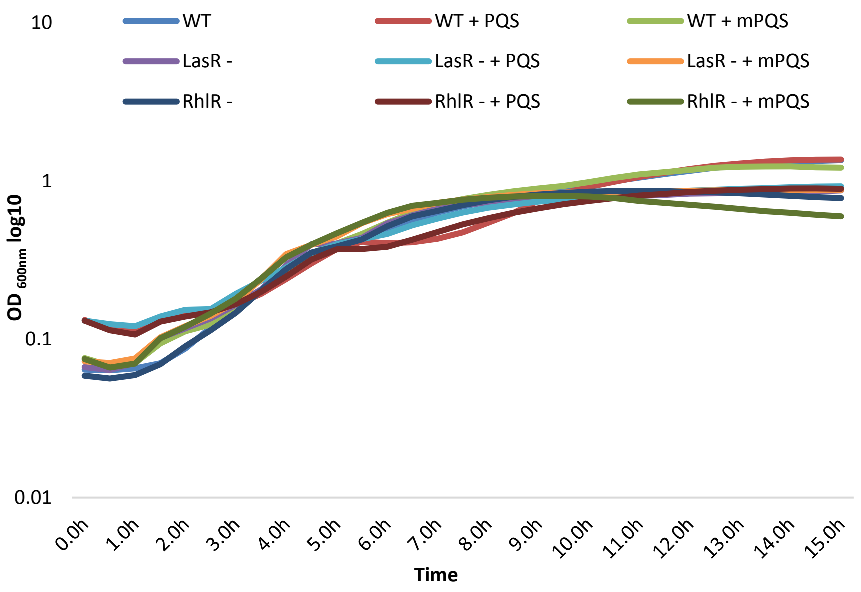

Supplementary Figure S8 – Growth dynamics of the strains from Figure 7. Mean of 3 biological replicates is shown.

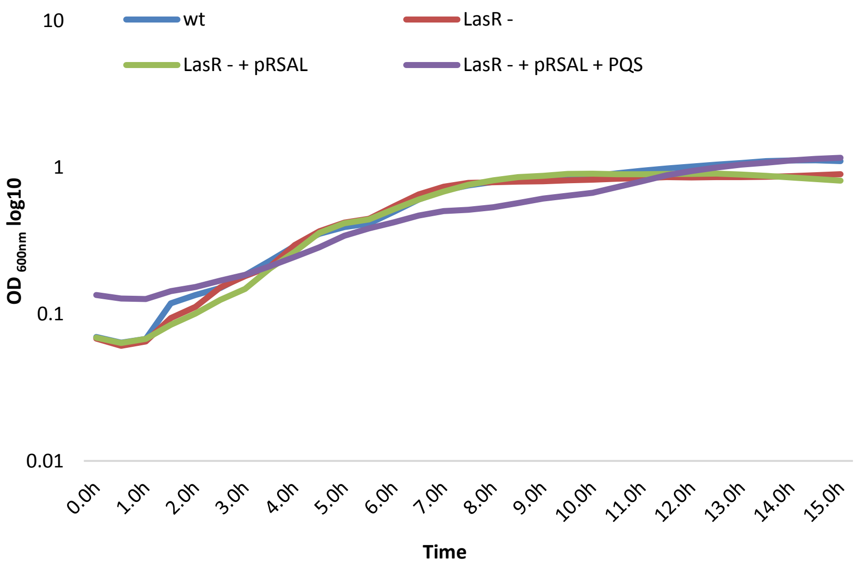

Supplementary Figure S9 – Growth dynamics of the strains from Figure 8. Mean of 3 biological replicates is shown.

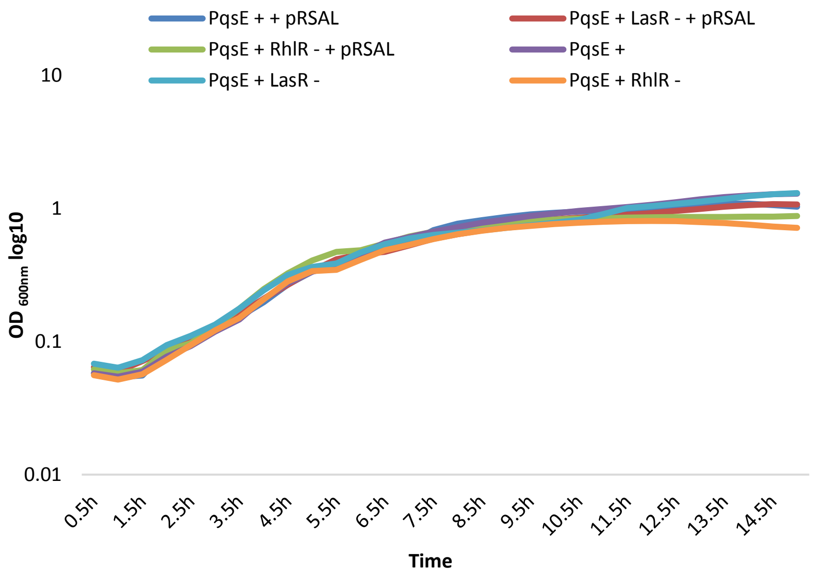

Supplementary Figure S10 – Growth dynamics of the strains from Figure 9. Mean of 3 biological replicates is shown.

References

Atkinson, S., Chang, C.Y., Patrick, H.L., Buckley, C.M.F., Wang, Y., Sockett, R.E., et al. (2008). Functional interplay between the *Yersinia pseudotuberculosis* YpsRI and YtbRI quorum sensing systems modulates swimming motility by controlling expression of *flhDC* and *fliA*. *Molecular Microbiology* 69(1)**,** 137-151. doi: 10.1111/j.1365-2958.2008.06268.x.

Grant, S.G.N., Jessee, J., Bloom, F.R., and Hanahan, D. (1990). Differential plasmid rescue from transgenic mouse DNAs into *Escherichia coli* methylation-restriction mutants. *Proceedings of the National Academy of Sciences of the United States of America* 87(12)**,** 4645-4649. doi: 10.1073/pnas.87.12.4645.

Hanahan, D. (1983). Studies on transformation of *Escherichia coli* with plasmids. *Journal of Molecular Biology* 166(4)**,** 557-580.

Harrison, F., Muruli, A., Higgins, S., and Diggle, S.P. (2014). Development of an ex vivo porcine lung model for studying growth, virulence, and signaling of *Pseudomonas aeruginosa*. *Infection and Immunity* 82(8)**,** 3312-3323. doi: 10.1128/iai.01554-14.

Heeb, S., Blumer, C., and Haas, D. (2002). Regulatory RNA as mediator in GacA/RsmA-dependent global control of exoproduct formation in *Pseudomonas fluorescens* CHA0. *Journal of Bacteriology* 184(4)**,** 1046-1056. doi: 10.1128/jb.184.4.1046-1056.2002.

Hoang, T.T., Karkhoff-Schweizer, R.R., Kutchma, A.J., and Schweizer, H.P. (1998). A broad-host-range Flp-FRT recombination system for site-specific excision of chromosomally-located DNA sequences: application for isolation of unmarked *Pseudomonas aeruginosa* mutants. *Gene* 212(1)**,** 77-86. doi: 10.1016/s0378-1119(98)00130-9.

Hoang, T.T., Kutchma, A.J., Becher, A., and Schweizer, H.P. (2000). Integration-proficient plasmids for *Pseudomonas aeruginosa*: Site-specific integration and use for engineering of reporter and expression strains. *Plasmid* 43(1)**,** 59-72.

Milton, D.L., O'Toole, R., Horstedt, P., and Wolf-Watz, H. (1996). Flagellin A is essential for the virulence of *Vibrio anguillarum*. *J Bacteriol* 178(5)**,** 1310-1319.
Rampioni, G., Pustelny, C., Fletcher, M.P., Wright, V.J., Bruce, M., Rumbaugh, K.P., et al. (2010). Transcriptomic analysis reveals a global alkyl-quinolone-independent regulatory role for PqsE in facilitating the environmental adaptation of *Pseudomonas aeruginosa* to plant and animal hosts. *Environmental Microbiology* 12(6)**,** 1659-1673. doi: 10.1111/j.1462-2920.2010.02214.x.
